# Three Genome Sequences of *Legionella pneumophila* subsp. *pascullei* Associated with Colonization of a Health Care Facility

**DOI:** 10.1128/genomeA.00335-16

**Published:** 2016-05-05

**Authors:** Natalia A. Kozak-Muiznieks, Shatavia S. Morrison, Scott Sammons, Lori A. Rowe, Mili Sheth, Michael Frace, Claressa E. Lucas, Vladimir N. Loparev, Brian H. Raphael, Jonas M. Winchell

**Affiliations:** a Division of Bacterial Diseases, National Center for Immunization and Respiratory Diseases, Centers for Disease Control and Prevention, Atlanta, Georgia, USA; b Biotechnology Core Facility Branch, Division of Scientific Resources, National Center for Emerging and Zoonotic Infectious Diseases, Centers for Disease Control and Prevention, Atlanta, Georgia, USA

## Abstract

Here, we report the complete genome sequences of three *Legionella pneumophila* subsp. *pascullei* strains (including both serogroup 1 and 5 strains) that were found in the same health care facility in 1982 and 2012.

## GENOME ANNOUNCEMENT

*Legionella pneumophila* is the major *Legionella* species responsible for causing severe pneumonia called Legionnaires’ disease (LD) (1). *L. pneumophila* is proposed to be composed of 3 subspecies: *pneumophila*, *fraseri*, and *pascullei* (2). Here, we report complete genome sequences of three *L. pneumophila* subsp. *pascullei* strains—D-7119, D-7158, and F-4185—all of which were associated with health care facility A (HCF-A) located in Pittsburgh, Pennsylvania, USA. Strain D-7158 (MICU-B, ATCC 33735) is an environmental isolate obtained from a tap-water sample in the medical intensive care unit of HCF-A in 1982 (3, 4). It belongs to serogroup 5 (Lp5) and sequence type (ST) 1335. Strains D-7119 and F-4185 are clinical and environmental isolates, respectively, associated with an LD outbreak at HCF-A that took place in 2012 (5). They both belong to serogroup 1 (Lp1) and ST 1395. D-7119 was isolated from sputum of an 86-year-old male patient in August 2012. F-4185 was grown from a bulk-water sample collected in the operating theater of HCF-A in November 2012.

Each isolate was sequenced using the Illumina MiSeq (San Diego, CA, USA) and Pacific Biosciences RSII (Menlo Park, CA, USA) sequencing platforms. An average of 5,000,000 Illumina sequencing reads and 139,728 PacBio reads were generated to construct a single contig for each isolate. Initially, we assembled PacBio data into a single-contig sequence using the SMRT Analysis portal version 2.3. The genome structure of these sequences was verified with optical maps, and Illumina reads were mapped to the PacBio sequence to ensure nucleotide accuracy, >99.9%. The genome sizes were 3,376,636 bp (D-7119), 3,458,918 bp (D-7158), and 3,385,087 bp (F-4185). PROKKA version 1.8 predicted between 2,988 (F-4185) and 3,064 (D-7158) coding sequences (6). Each isolate contained 43 predicted tRNAs. A 16S sequence comparison revealed that these isolates were more closely related to subspecies *pascullei* than to subspecies *pneumophila*. Average nucleotide identity calculations (7) further support the closer relationship for these strains to subspecies *pascullei* (~99.89%) than to *pneumophila* (~90.76%) or *fraseri* (~93.56%). During analysis of these completely closed genomes, we identified two large genomic islands that were present only in D-7158. The majority of genes within the 36.8-kb island A were biosynthetic, whereas the 51.9-kb island B contains several phage and mobilization-related genes. We identified 47 Lp5-specific genes and 16 Lp1-specific genes, 12 of which belonged to the LPS biosynthesis region. The content of specific LPS biosynthesis-encoding genes in Lp5 strain D-7158 was 100% identical to those in the genome of the Lp5 type strain Dallas-1E, and different from that in Lp1 D-7119, F-4185, or an Lp1 reference strain Philadelphia-1 (NC_002942.1).

These genomes will be helpful to the *Legionella* research community for studying rare representatives of *pascullei* subspecies and to better understand the molecular basis for serogroup differences among closely related strains. Moreover, these data can be used for developing a method for quick identification of Lp5 strains by detecting the LPS biosynthesis gene content.

### Nucleotide sequence accession numbers.

The genome sequences described in this paper have been deposited at DDBJ/EMBL/GenBank under the accession numbers CP014257 (D-7119), CP014256 (D-7158), and CP014255 (F-4185).
